# Molecular genetic diversity and bioinformatic analysis of *Leucocytozoon sabrazesi* based on the mitochondrial genes *cytb*, *coxI* and *coxIII* and co-infection of *Plasmodium* spp.

**DOI:** 10.1051/parasite/2022022

**Published:** 2022-04-27

**Authors:** Pornpiroon Nooroong, Amaya Watthanadirek, Sutthida Minsakorn, Napassorn Poolsawat, Witchuta Junsiri, Nitipon Srionrod, Siriphan Sangchuai, Runglawan Chawengkirttikul, Panat Anuracpreeda

**Affiliations:** 1 Parasitology Research Laboratory (PRL), Institute of Molecular Biosciences, Mahidol University Nakhon Pathom 73170 Thailand; 2 Department of Parasitology, Faculty of Medicine Siriraj Hospital, Mahidol University Bangkok 10700 Thailand; 3 Department of Microbiology, Faculty of Science, Mahidol University Bangkok 10400 Thailand

**Keywords:** *Leucocytozoon sabrazesi*, *Plasmodium* spp., Co-infection, Mitochondrial genes, Genetic diversity, Chickens, Thailand

## Abstract

*Leucocytozoon sabrazesi* is an intracellular haemoprotozoan parasite responsible for leucocytozoonosis, which is transmitted by insect vectors and affects chickens in tropical and subtropical areas in many countries. It causes huge economic losses due to decreased meat and egg production. In the present study, we used nested PCR to determine the genetic diversity of *L. sabrazesi* based on the *cytb, coxI, coxIII* and concatenated genes in chickens in Thailand. In addition, we found co-infections between *L. sabrazesi* and *Plasmodium* spp. (*P. gallinaceum* or *P. juxtanucleare*) in chickens that were not identified by microscopic examination of blood smears. The phylogenetic analysis indicated that *L. sabrazesi cytb* and *coxIII* genes were conserved with similarity ranging from 99.9 to 100% and 98 to 100%, respectively whereas the *coxI* gene was diverse, with similarities ranging from 97 to 100%. These findings ascertained the nucleotide analysis of the *cytb, coxI, coxIII* and concatenated sequences in which 4, 8, 10 and 9 haplotypes were found, respectively. In addition, it was found that the large number of synonymous substitutions and conservative amino acid replacements in these mitochondrial genes occurred by non-synonymous substitution. The evolutionary analysis of the *K*_a_/*K*_s_ ratio supported purifying selection and the negative values of both Fu’s *F*s and Tajima’s *D* indicate selective sweep especially for the *coxI* gene. The entropy and Simplot analysis showed that the genetic variation in populations of *Plasmodium* spp. was higher than in *Leucocytozoon.* Hence, the nucleotide sequences of three mitochondrial genes could reflect the evolutionary analysis and geographic distribution of this protozoan population that switches hosts during its life cycle.

## Abstract (fr)

*Leucocytozoon sabrazesi* est le parasite hémoprotozoaire intracellulaire responsable de la leucocytozoonose, qui est transmise par des insectes vecteurs et affecte les poulets dans les zones tropicales et subtropicales de nombreux pays. Il provoque d’énormes pertes économiques en raison de la diminution de la production de viande et d’œufs. Dans la présente étude, nous avons utilisé la PCR nichée pour déterminer la diversité génétique de *L. sabrazesi* sur la base des gènes cytb, coxI, coxIII et concaténés chez des poulets en Thaïlande. De plus, nous avons trouvé des co-infections entre *L. sabrazesi* et *Plasmodium* spp. (*P. gallinaceum* ou *P. juxtanucleare*) chez des poulets, qui n’ont pas été identifiées par l’examen microscopique de frottis sanguins. L’analyse phylogénétique a indiqué que les gènes cytb et coxIII de *L. sabrazesi* étaient conservés avec une similarité allant respectivement de 99,9 à 100 % et de 98 à 100 %, alors que le gène coxI était diversifié, avec des similarités allant de 97 à 100 %. Ces découvertes ont confirmé l’analyse des nucléotides des séquences cytb, coxI, coxIII et concaténées dans lesquelles 4, 8, 10 et 9 haplotypes ont été trouvés, respectivement. De plus, il a été constaté que le grand nombre de substitutions synonymes et de remplacements conservateurs d’acides aminés dans ces gènes mitochondriaux se produisaient par substitution non synonyme. L’analyse évolutive du rapport *K*_a_/*K*_s_ a soutenu la sélection purificatrice et les valeurs négatives des Fs de Fu et D de Tajima indiquent un balayage sélectif, en particulier pour le gène coxI. L’entropie et l’analyse Simplot ont montré que la variation génétique de la population de *Plasmodium* spp. était plus élevée que pour *Leucocytozoon*. Par conséquent, les séquences nucléotidiques de trois gènes mitochondriaux pourraient refléter l’analyse évolutive et la répartition géographique de cette population de protozoaires qui changent d’hôte au cours de leur cycle de vie.

## Introduction

*Leucocytozoon sabrazesi* Mathis & Léger, 1911 is an important blood parasite belonging to the phylum Apicomplexa, which commonly infects a wide range of avian species. In addition, *L. sabrazesi* has frequently been reported in both fighting cocks (*Gallus gallus)* and domestic chickens (*Gallus gallus domesticus*) [2, 32, 41]. Both black fly (Simuliidae) and culicoides midges (Ceratopogonidae) act as potential vectors for *Leucocytozoon* transmission [20, 21, 36, 46–48]. *Leucocytozoon sabrazesi* infections of domestic stock cause symptoms including lethargy, green feces, loss of appetite, anemia, and death. Further, infections are known to cause economic losses through increased chicken mortality and reduced egg production [3, 28, 35]. Notably, leucocytozoonosis or *Leucocytozoon* infection are reported in many kinds of birds around the world, including in Asia (Thailand), Africa, Europe, and North America [6, 10, 39, 40, 45].

A conventional diagnosis of *Leucocytozoon* infection is based on microscopic examination of the gametocytes in Giemsa-stained blood smears of the infected chickens. Currently, polymerase chain reaction (PCR) may be more reliable and widely used to diagnose the infection and be supplemented by the standard parasitological method, especially in the laboratory for high sensitivity and specificity even when blood smears are negative with low parasitemia. Although the diversity of hemosporidian parasites has been demonstrated based on mitochondrial genes, such as cytochrome b (*cytb*), in ecological and evolutionary studies [4, 17], there is little information about the genetic diversity of *L. sabrazesi* isolates in Thailand with *Plasmodium* spp. co-infection when using mitochondrial genes (*cytb*, *coxI* and *coxIII*). Therefore, this study aimed to investigate the mitochondrial genetic diversity of *L. sabrazesi* and *Plasmodium spp.* coinfections in chickens in Thailand at these three loci, including phylogenetic and biogeographic relationships. In addition, the phylogenetic relationship, haplotype diversity, entropy, and geographic and evolutionary distribution among the isolates identified in this work and those from other countries are presented.

## Materials and methods

### Ethics statement

Experimentation on animals was carried out under the following approval and permit from the Animal Care and Use Committee (IMBMU-ACUC), Institute of Molecular Biosciences, Mahidol University, Thailand. All suitable international, national and/or institutional guidelines for animal care and use were followed. Also, we received consent to collect chicken blood samples at the animal farm.

### Blood sample collection

Thirty chickens (*Gallus gallus domesticus*) from the Bongti (14°04′20.8″N 98°59′50.1″E) and Tha Sao (14°10′27.2″N 99°07′15.6″E) districts in Kanchanaburi province, Thailand were collected via the brachial wing vein. The blood samples were kept in sterile 1.5-mL tubes containing lithium heparin to prevent coagulation, and stored at −80 °C until use.

### Ficoll density gradient centrifugation

For Giemsa-stained blood smears, elongated gametocytes of *Leucocytozoon sabrazesi* were detected in 30 chicken blood samples. The blood samples were diluted with 0.1 M phosphate-buffered saline (PBS), pH 7.4 and overlayed with Ficoll-Paque (Sigma-Aldrich, Burlington, MA, USA). They were centrifuged at 400 ×*g* for 30 min at 25 °C. The gametocytes were gently harvested by inserting the pipette directly through the upper layer and later washed twice in the PBS solution.

### *Leucocytozoon sabrazesi* DNA extraction

The genomic DNA of *L. sabrazesi* in blood samples was extracted by using an E.Z.N.A.^®^ Tissue DNA Kit (OMEGA Bio-Tek, Norcross, GA, USA) following the protocol of Watthanadirek et al. [42, 43] and Junsiri [22] with some modifications. Briefly, 250 μL of blood samples were mixed thoroughly with 25 μL of proteinase K solution, and incubated at 70 °C for 10 min. Then, 250 μL of absolute ethanol were added and all lysates transferred to the HiBind^®^ DNA Mini Column. The lysates were centrifuged at maximum speed for 1 min before adding 500 μL of HBC buffer. After adding 700 μL of DNA washing buffer, the genomic material was eluted with 50 μL of elution buffer. Finally, the extracted DNA solutions were stored at −20 °C until further use.

### Molecular amplification of *L. sabrazesi* DNA

The *cytb*, *coxI*, and *coxIII* genes of *L. sabrazesi* were amplified by nested PCR using the specific primers: Hemo_cytbF (5′–CATATATTAAGAGAATTATGGAG–3′) and Hemo_cytbR (5′–ATAAAATGYTAAGAAATACCATTC–3′) (GenBank accession number AB299369) for the first step of amplification. At the second step of amplification, Ls_cytbF (5′–CACC TAATCACATGGGTTTGTGGA–3′) and Ls_cytbR (5′–GCTTTGGGCTAAGAATAATACC–3′) for the *cytb* gene, PgCoxIF (5′–CACCGCGTACTTTGGACCGAAAAA–3′) and PgCoxIR (5′–CATCCAGTACCACCACCAAA–3′) for the *coxI* gene, as well as CoxIII F (5′–CACCTAA CAT TCT ACA TGA TGT AGT–3′) and CoxIII R (5′–GTAAAAGCACACTTATCTAG–3′) for the *coxIII* gene were used in this study. The 4 base sequences (CACC) were added to the 5′ end of forward primers with overhang sequence (GTGG) in a pET100/D-TOPO^®^ vector (Invitrogen, Waltham, MA, USA) to certify a cloning direction. The PCR reaction mixture contained 10× Standard *Taq* Reaction Buffer, 10 mM of each deoxynucleotide triphosphate (dNTPs), 10 μM of forward and reverse primers, 0.625 U of *Taq* DNA polymerase (NEB, UK), RNase-free water, and 1 μg of DNA template. The thermal cycling was performed in Mastercycler^®^ nexus Thermal Cycler (Germany) with 95 °C for 2 min, followed by 35 cycles of 95 °C for 30 s, 55 °C for 30 s, 68 °C for 1 min, and then 68 °C for 5 min. The RNase-free water and confirmed *L. sabrazesi* DNA samples were used as negative and positive controls, respectively. The PCR products were separated by 1% agarose gel electrophoresis and stained with SYBR green fluorescence dye, then visualized under ultraviolet light. The positive samples were purified using a PureDireX PCR Clean-Up & Gel Extraction Kit (Bio-Helix Co., Taiwan).

### Cloning and sequencing of the *L. sabrazesi* cytb, coxI and coxIII genes

The PCR products were purified using a PureDireX PCR Clean-Up & Gel Extraction Kit. The 5′ blunt end of purified PCR products was ligated into a pET100/D-TOPO^®^ vector (Invitrogen Life Technologies, Carlsbad, CA, USA). The ligation products were heat-shocked and transformed into chemically competent *Escherichia coli* host strain TOP10 cells (Invitrogen Life Technologies). The transformed *E. coli* cultures were spread on Luria-Bertani (LB) agar plates containing 100 μg of ampicillin and incubated at 37 °C for 16 h. The positive bacterial colonies were picked and cultured in LB media containing ampicillin with shaking at 37 °C for 16 h. The plasmids were extracted from bacterial cultures using an AxyPrep Plasmid Miniprep Kit (Axygen Bioscience, Union City, CA, USA) before sequencing.

### Sequence and *in silico* analysis

The presence of *cytb, coxI* and *coxIII* inserts was confirmed by Sanger sequencing. All sequences were submitted and deposited in the National Center for Biotechnology Information (NCBI) GenBank database. The sequences were also analyzed by BLAST (https://blast.ncbi.nlm.nih.gov). All nucleotide and amino acid sequences were analyzed by the computer programs MEGA 7.0.26 [24] and Jalview [10]. For nucleic acid substitution analysis, nucleotide diversity was determined using DnaSP software, V.6.0 [26]. All base substitutions were determined as synonymous and nonsynonymous substitutions in nucleotides and amino acid sequences were assessed using PROVEAN analysis [9] as compensation of physicochemical properties of amino acid replacement. In addition, the haplotype analysis was determined through DnaSP software, V.6.0 [26] before visualization of the mutational occurrence of haplotypes from different geographic distribution, and the relationships among haplotypes were visualized with a TCS network in the popART program [25].

### Multiple sequence alignment and phylogenetic analysis

The *cytb, coxI* and *coxIII* sequences were employed for sequence alignment and phylogenetic analysis. Multiple sequence alignments were conducted with the MUSCLE algorithm [12]. All aligned DNA sequences were used to construct the molecular phylogenetic trees using neighbour-joining (NJ), maximum likelihood (ML), maximum parsimony (MP) and Bayesian analysis (BA) [19]. The reliability of the internal branching pattern of the phylogenetic tree was determined in each clade by statistical calculation of 1000 replicates using the bootstrapping method [13] and MrBayes program for posterior probability. The evolutionary distances were evaluated by the Kimura 2-parameter method [23]. Similarity (as a percentage) was also analyzed by using a sequence identity matrix in BioEdit software V.7.0.5.3 [16].

### Entropy analysis

The entropy values for nucleotide and amino acid variation were assessed with Shannon’s entropy (*H*(*x*)) plot method in BioEdit software, V.7.0.5.3 [1, 16].

## Results

### Determination of *L. sabrazesi* mitochondrial gene sequences

The DNA sequences of *L. sabrazesi cytb, coxI* and *coxIII* were partially amplified by nested PCR. The quality of PCR products was evaluated by the ratio of optical density (OD_260/280_) of 1.8–2.0, which showed no contamination of the products. The lengths of *cytb, coxI* and *coxIII* sequences Thailand strain were 248, 588 and 294 bp, respectively. All DNA sequences of *L. sabrazesi* investigated in this study were submitted and deposited in the NCBI GenBank database (https://www.ncbi.nlm.nih.gov/genbank/) under accession numbers MZ634375 to MZ634390 for the *cytb* gene, MZ634391 to MZ634403 for the *coxI* gene, and MZ634404 to MZ634417 for the *coxIII* gene (Table 1).

**Table 1:** The *L. sabrazesi* and *Plasmodium* spp. mitochondrial nucleotide sequences amplified in Thailand deposited in GenBank.

Province	Districts	Animal ID	GenBank accession numbers			
			*L. sabrazesi*			*Plasmodium* spp.
			*cytb*	*coxI*	*coxIII*	*coxI*
Kanchanaburi	Bongti (14°04′20.8″N 98°59′50.1″E)	PBMC1-1	MZ634375	MZ634391	MZ634404	
		PBMC1-3	MZ634376	MZ634392		
		PBMC1-6	MZ634377	MZ634393	MZ634405	
		PBMC1-13	MZ634378	MZ634394		
		PBMC1-18	MZ634379		MZ634406	MZ634402
		PBMC1-19	MZ634380		MZ634407	
		PBMC1-20	MZ634381		MZ634408	MZ634403
		PBMC1-21	MZ634382		MZ634409	
		PBMC1-23	MZ634383		MZ634410	
	Tha Sao (14°10′27.2″N 99°07′15.6″E)	PBMC1	MZ634384	MZ634395	MZ634411	
		PBMC4	MZ634385	MZ634396	MZ634412	
		PBMC5	MZ634386	MZ634397	MZ634413	
		PBMC6	MZ634387		MZ634414	MZ634399
		PBMC8	MZ634388		MZ634415	MZ634400
		PBMC10	MZ634389		MZ634416	MZ634401
		PBMC15	MZ634390		MZ634417	MZ634398

### Phylogenetic analysis

**Fig. 1:**
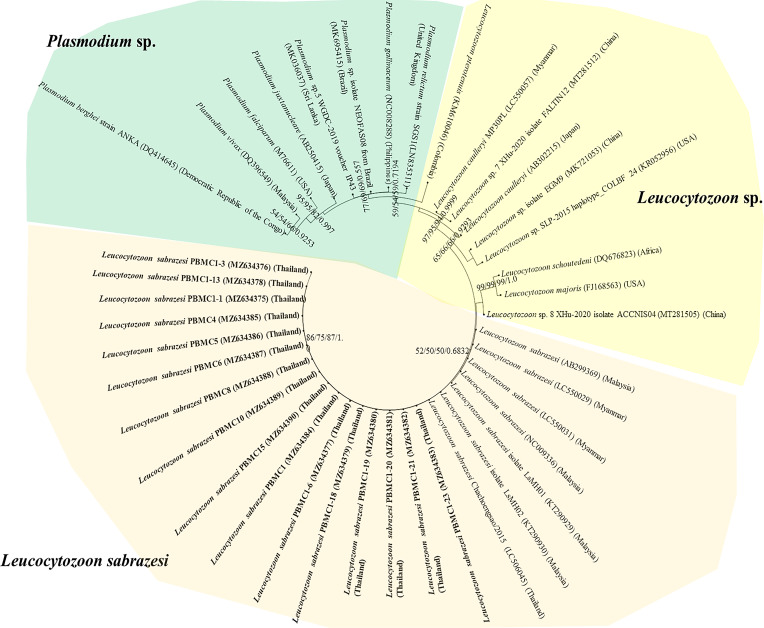
Phylogenetic tree of the *cytb* gene sequences in this study (bold face) and those taken from GenBank. The boostrap values calculated from NJ, ML, MP and BA are labeled on each branch.

**Fig. 2:**
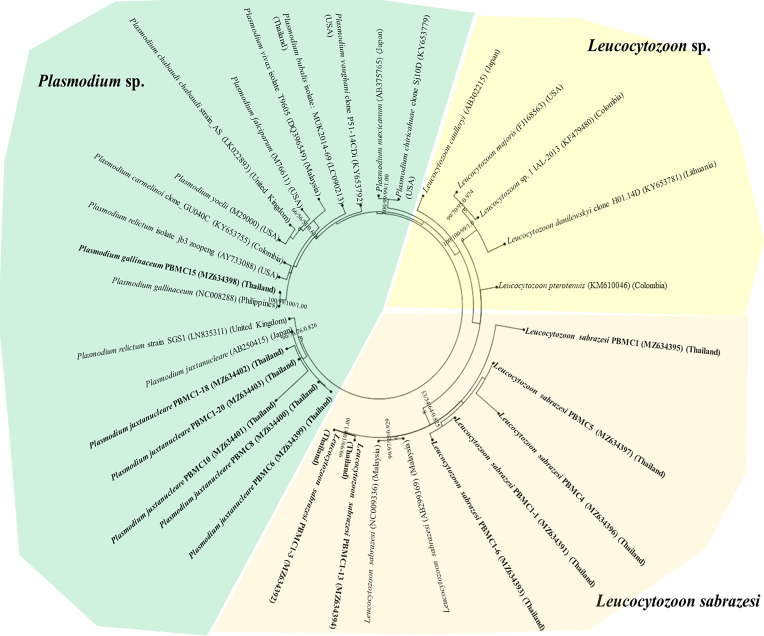
Phylogenetic tree of the *coxI* gene sequences in this study (bold face) and those obtained from GenBank. The boostrap values calculated from NJ, ML, MP and BA are labeled on each branch.

**Fig. 3:**
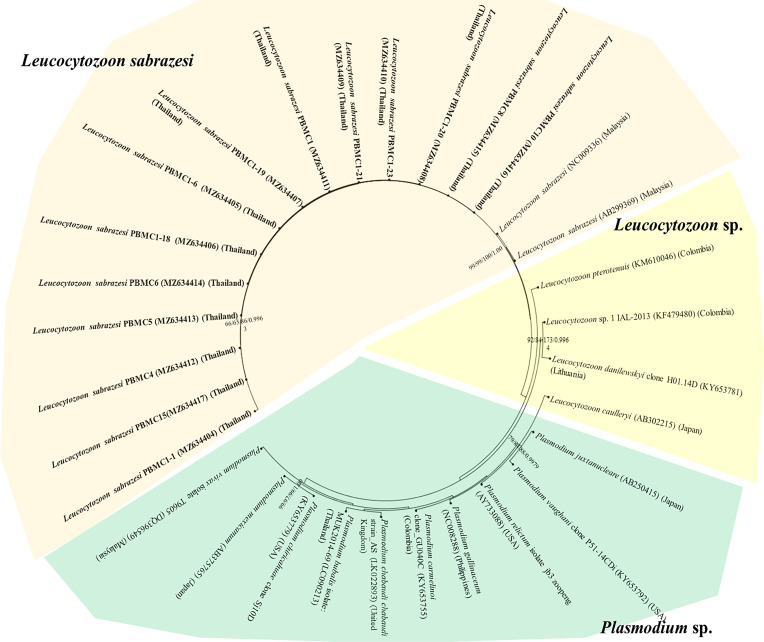
Phylogenetic tree of the *coxIII* gene sequences in this study (bold face) and those taken from GenBank.The boostrap values calculated from NJ, ML, MP and BA are labeled on each branch.

**Fig. 4:**
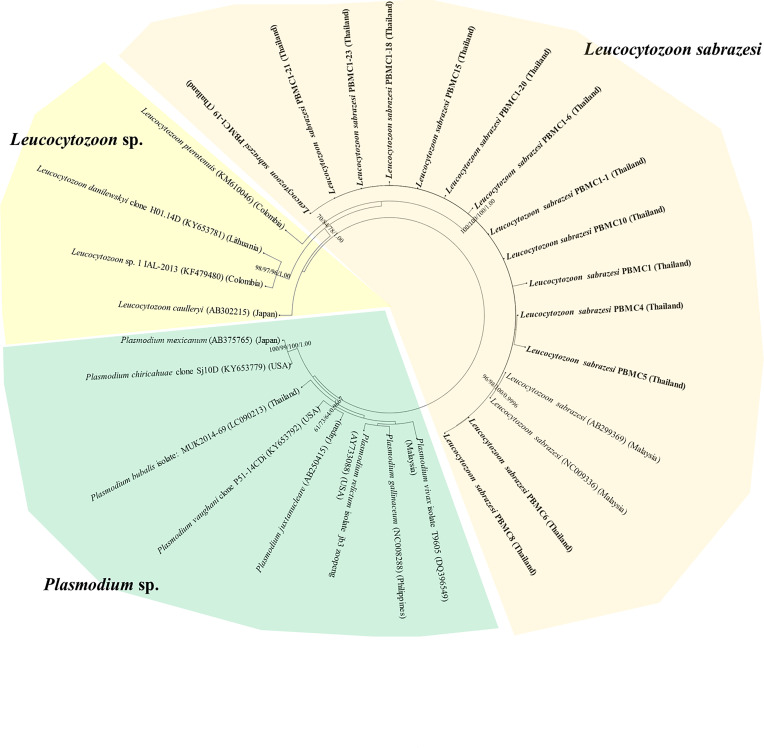
Phylogenetic tree of the concatenated gene sequences in this study (bold face) and those obtained from GenBank. The boostrap values calculated from NJ, ML, MP and BA are labeled on each branch.

The *L. sabrazesi cytb* sequences obtained in this work were aligned with other sequences retrieved from GenBank including sequences from Thailand, Malaysia, Myanmar, China, USA, Uganda, Congo, Sri Lanka, Brazil, Philippines, UK and Japan. Our sequences detected in this study were positioned in the same clade as *L. sabrazesi* (Fig. 1). The Thailand *coxI* sequences were determined in the different clades in the phylogenetic tree together with other sequences of *P. gallinaceum* and *P. juxtanucleare* (Fig. 2), while the phylogenetic tree constructed from *coxIII* sequences was positioned in the same clade as *L. sabrazesi* (Fig. 3). Not only the phylogenetic tree constructed from each mitochondrial sequence, but also the concatenated genes from all mitochondrial sequences were used to construct the phylogenetic tree, which showed that our sequences were grouped and positioned in the same clade as *L. sabrazesi* (Fig. 4). Moreover, the reliability of bootstrap frequencies and Bayesian posterior probabilities of all phylogenies are displayed with the highest values on each branch.

### Similarity analysis

All DNA samples from chickens were positive for all three mitochondrial genes. The similarity among the Thailand *cytb, coxI* and *coxIII* sequences taken in this study was 99.9–100%, 97–100% and 98–100%, respectively (Tables S1–S3), while the similarity of those compared only between *Plasmodium* sequences obtained from GenBank by BLAST was 85–100%, 86–100% and 84–100%, respectively (Tables S1–S3). Interestingly, one sequence in this study showed 100% similarity co-infection of *L. sabrazesi* and *P. gallinaceum* (Tables 1 and S2)*.* As well, our two sequences exhibited 100% similarity of co-infection of *L. sabrazesi* and *P. juxtanucleare* (Tables 1 and S2)*.* For amino acid sequencing of *L. sabrazesi*, the similarity among the Thailand *cytb, coxI* and *coxIII* sequences taken in this study was 98–100%, 97–100% and 96–100%, respectively, whereas the similarity of these compared with other sequences obtained from GenBank by BLAST was 74–100%, 60–99% and 60–100%, respectively (Tables S4–S6).

### Entropy analysis

**Fig. 5:**
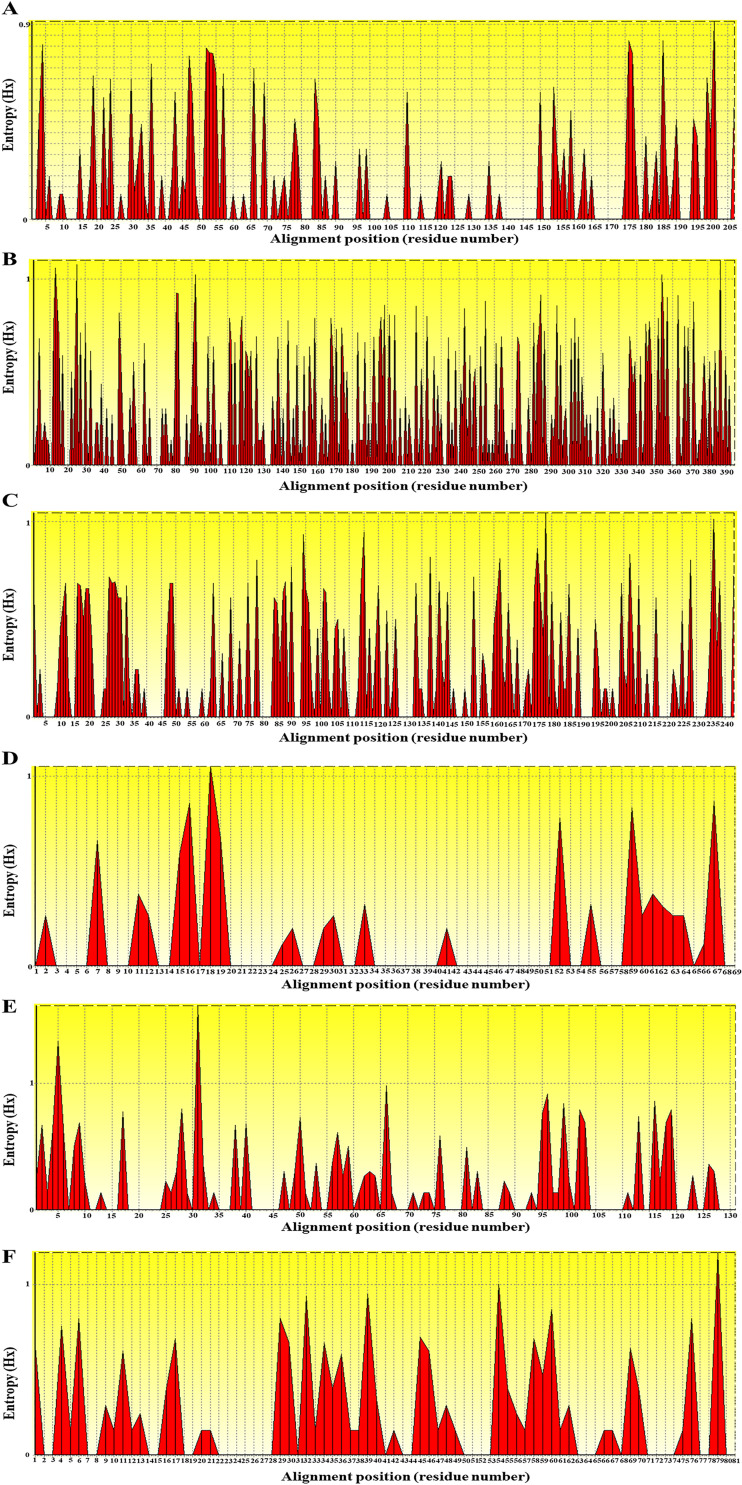
Entropy analysis of *L. sabrazesi cytb*, *coxI* and *coxIII* gene sequences. Entropy plot of multiple nucleic acid sequence alignment of the *cytb* (A), *coxI* (B) and *coxIII* (C) genes. The red peaks indicate the high variation at each position of the nucleic acid sequences. Entropy plot of multiple amino acid sequence alignment of CYTb (D), COXI (E) and COXIII (F). The red peaks indicate the high variation at each position of amino acid sequences.

**Supplementary Figure 1.**
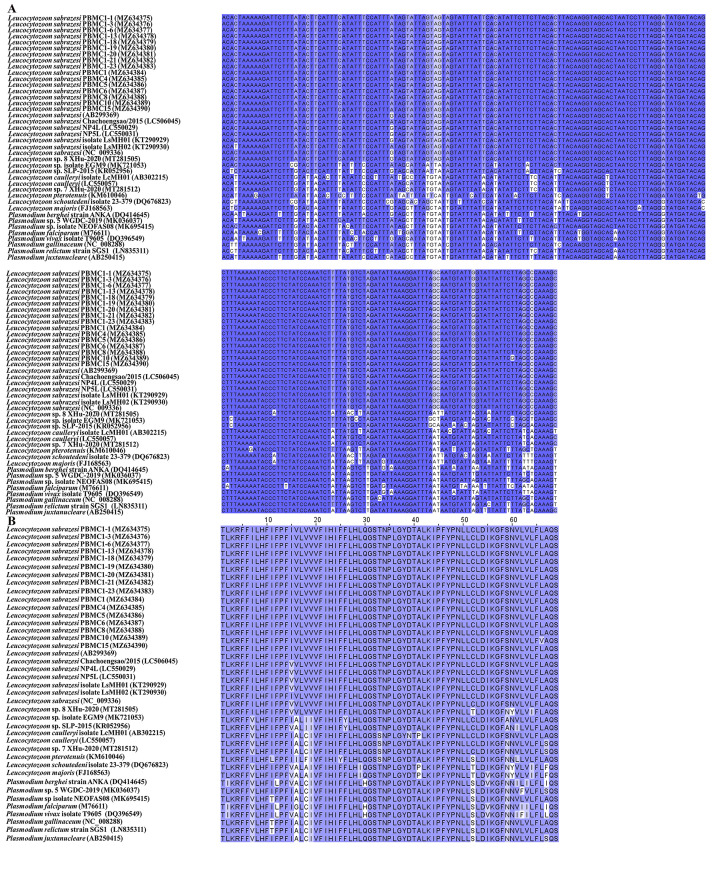
Alignment of nucleic acid sequences of the *cytb* gene among *L. sabrazesi* and *Plasmodium* spp. The highest similarity of nucleotide positions is represented with dark blue color, while white color represents the least similarity of each nucleic acid position (A). Multiple amino acid sequence alignment of CYTb protein among *L. sabrazesi* and *Plasmodium* spp. The highest similarity of physicochemical properties (BLOSUM score 62) of each amino acid position is represented with blue color, while white color represents the least similarity of each amino acid position (B).

**Supplementary Figure 2:**
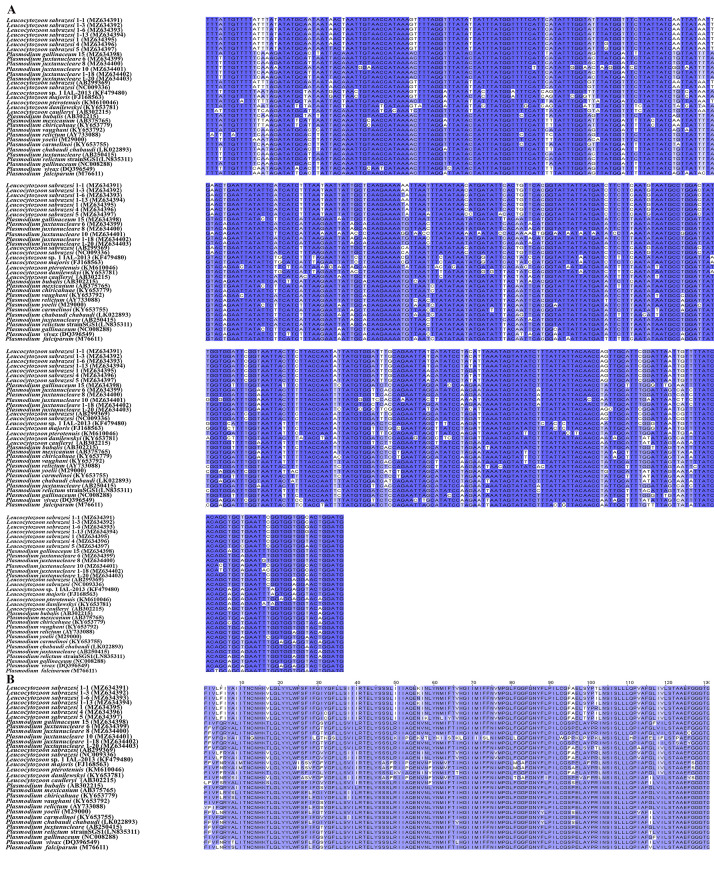
Alignment of nucleic acid sequences of *coxI* gene among *L. sabrazesi* and *Plasmodium* sp.p The highest similarity of nucleotide positions is represented with dark blue color, while white color represents the least similarity of each nucleic acid position (A). Multiple amino acid sequence alignment of COXI protein among *L. sabrazesi* and *Plasmodium* spp. The highest similarity of physicochemical properties (BLOSUM score 62) of each amino acid position is represented with blue color, while white color represents the least similarity of each amino acid position (B).

**Supplementary Figure 3:**
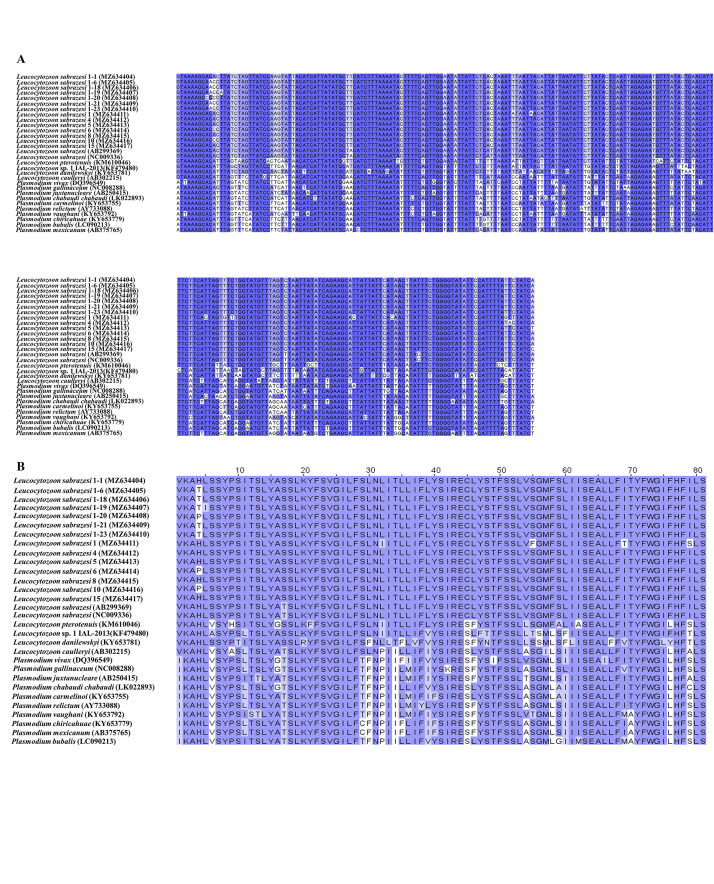
Alignment of nucleic acid sequences of *coxIII* gene among *L. sabrazesi* and *Plasmodium* sp. The highest similarity of nucleotide positions is representd with dark blue color, while white color represents the least similarity of each nucleic acid position(A). Multiple amino acid sequence alignment of COXIII protein among *L. sabrazesi* and *Plasmodium* spp. The highest similarity of physicochemical properties (BLOSUM score 62) of each amino acid position is represented with blue color, while white color represents the least similarity of each amino acid position (B).

The similarity analysis from Simplot showed higher nucleotide variation in *Plasmodium* spp. than in *L. sabrazesi.* The entropy analysis of the *cytb, coxI* and *coxIII* genes showed more variation of nucleic acid sequences than amino acid sequences. To analyze nucleic acid entropy, *cytb, coxI* and *coxIII* sequences showed 81 peaks with entropy values ranging from 0.11691 to 0.93764, 174 peaks with entropy values ranging from 0.13579 to 1.06709, and 125 peaks with entropy values ranging from 0.14614 to 0.94469, respectively. Entropy analysis of amino acid sequences exhibited that the charts showed 24 peaks with entropy values ranging from 0.11691 to 1.05331 for *cytb*, 62 peaks with entropy values ranging from 0.13579 to 1.61397 for *coxI*, and 46 peaks with entropy values ranging from 0.14614 to 1.18722 for *coxIII* (Fig. 5). The *coxI* gene was found to be more diverse than *cytb* and *coxIII* and this is consistent with multiple sequence alignment which showed more similarity among amino acid sequences than nucleic acid sequences (Supplementary Figs. 1–3). The nucleic acid variation from multiple sequence alignment correlated to high nucleic acid diversity in the *coxI* gene caused by nucleic acid sequences of *L. sabrazesi.* Besides the *coxI* gene, both the *cytb* and *coxIII* genes exhibited higher genetic diversity in *Plasmodium* spp. than in *L. sabrazesi* (Tables 2 and 3)*.*

**Table 2:** Comparison of nucleotide sequence analyses of three mitochondrial and concatenated genes of *Leucocytozoon* spp*.* and *Plasmodium* spp. as detected in chicken samples in Thailand and other countries.

Comparison	*cytb*		*coxI*		*coxIII*		Concatenated gene	
	*Leucocytozoon* spp.	*Plasmodium* spp.	*Leucocytozoon* spp.	*Plasmodium* spp.	*Leucocytozoon* spp.	*Plasmodium* spp.	*Leucocytozoon* spp.	*Plasmodium* spp.
No. of polymorphic sites	65	46	228	104	93	73	149	123
No. of mutations	78	52	304	122	102	84	162	136
Nucleotide difference (k)	13.153	18.857	64.332	38	17.146	28.927	26.83	48.139
Nucleotide diversity (Pi)	0.06354	0.0911	0.162286	0.0962	0.07056	0.11904	0.06002	0.1079
No. of haplotypes (h)	12	8	19	13	14	10	13	8
	*cytb*		*coxI*		*coxIII*		Concatenated gene	
No. of haplotypes	20		32		24		21	
Haplotype diversity (Hd)	0.832		0.998		0.979		0.966	
Fu’s *F*s statistic	−0.09404		−1.39769		−0.6347		−0.40481	
Tajima’s *D*	−0.76952		−1.05145		−0.11697		−0.29339	
*K*_a_	0.07327		0.09485		0.17167		0.12331	
*K*_s_	0.56524		0.56374		0.75719		0.68005	
*K*_a_/*K*_s_ ratio	0.13		0.168		0.227		0.181	
A (%)	33.03		31.23		46.12		31.93	
T/U (%)	40.58		42.66		31.12		43.59	
G (%)	10.29		14.01		13.62		9.75	
C (%)	16.1		12.1		9.14		14.74	

**Table 3:** Polymorphism and genetic diversity of the three mitochondrial and concatenated genes of *Leucocytozoon* spp. *and Plasmodium* spp. as detected in chicken samples in Thailand and other countries.

Genes	Size (bp)	*N*	VS	GC%	Dh (mean ± SD)	*π* (mean ± SD)	*K*
Sequence of *Leucocytozoon* spp.							
*cytb*	248	32	65	27.54	0.736 + 0.078	0.06354 + 0.01628	13.153
*coxI*	588	20	228	26.53	0.995 + 0.018	0.162286 + 0.01514	64.332
*coxIII*	294	19	93	23.65	0.83 + 0.085	0.07056 + 0.02758	17.146
Concatenated gene	1130	20	149	26.05	0.932 + 0.039	0.06002 + 0.02524	26.83
Sequence of *Plasmodium* spp.							
*cytb*	248	8	46	24.09	1 + 0.063	0.0911 + 0.01155	18.857
*coxI*	588	13	104	25.33	1 + 0.030	0.0962 + 0.00702	38
*coxIII*	294	11	73	21.43	1 + 0.039	0.11904 + 0.00972	28.927
Concatenated gene	1130	8	123	23.91	1 + 0.063	0.1079 + 0.01084	48.139

*N* = number of analyzed sequences; VS = number of variable sites; GC = G × C content; Dh = diversity of haplotypes; SD = standard deviation; *π* = nucleotide diversity (per site); *K* = average number of nucleotide differences.

### Nucleic acid substitution analysis

**Fig. 6:**
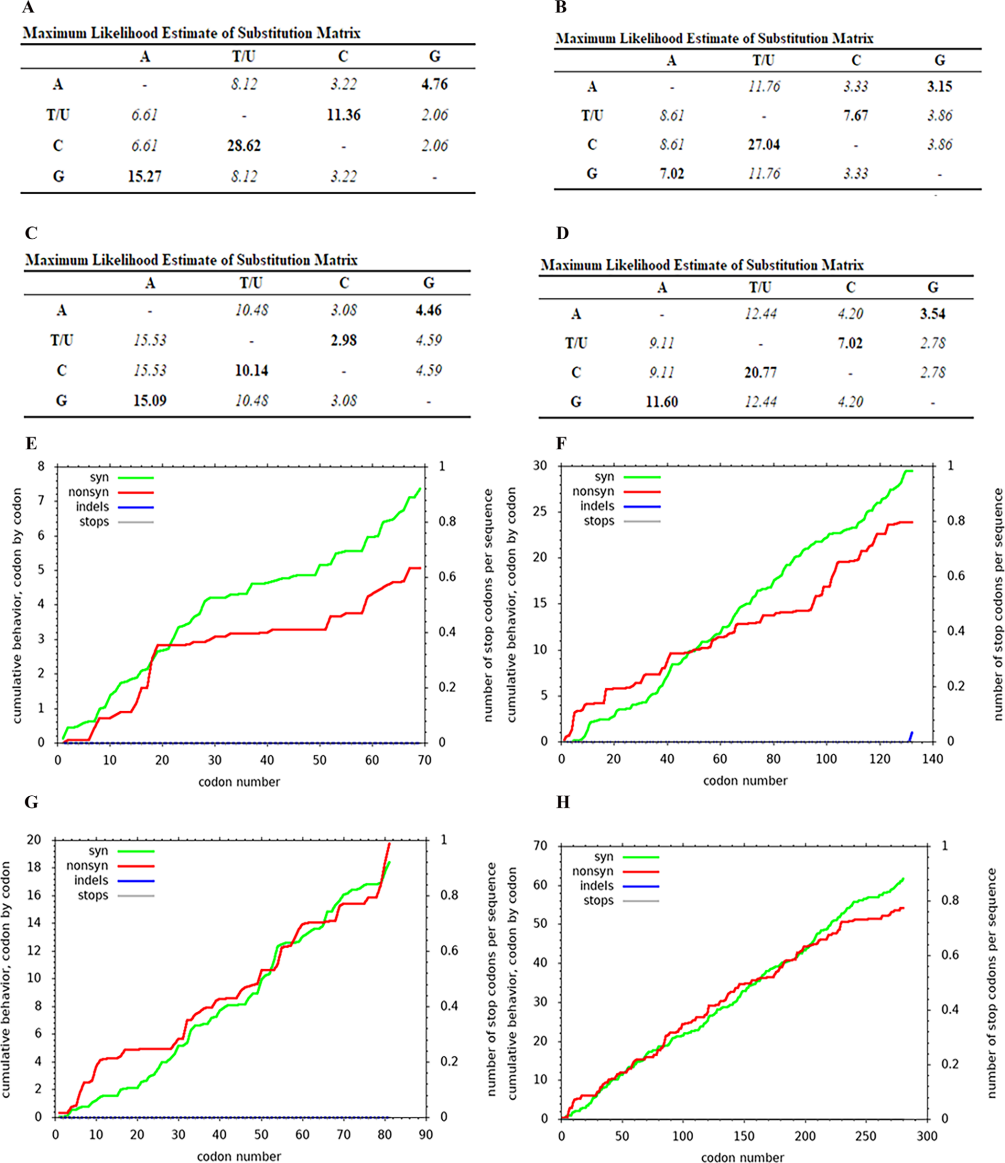
Nucleic acid substitution rate and base composition of *cytb, coxI, coxIII* and concatenated sequences among *Leucocytozoon* spp. and *Plasmodium* spp. Tables showing the transition and transversion from nucleotide substitution in *cytb* (A), *coxI* (B.), *coxIII* (C) and concatenated (D) genes. Graph incidating the synonymous and non-synonymous substitutions of *cytb* (E), *coxI* (F), *coxIII* (G) and concatenated (H) genes of *Leucocytozoon* spp. and *Plasmodium* spp.

Each nucleic acid substitution of *cytb, coxI* and *coxIII* was validated as transition from purine to purine and from pyrimidine to pyrimidine. In addition, the percentage of base composition of these genes indicated the number of A and T bases greater than G and C contents. However, most base substitutions were indicated as the synonymous substitutions (Fig. 6). Moreover, the synonymous frequency (*K*_s_) of these genes was higher than non-synonymous frequency values (*K*_a_). The *K*_a_/*K*_s_ ratios of *cytb, coxI, coxIII* and concatenated genes were 0.13, 0.168, 0.227 and 0.181, respectively (Table 2). While all results of the evolutionary estimation of Tajima’s *D* values exhibited minus values, only *coxI* showed statistical significance, which determined an excess of low frequency polymorphisms relative to expectations under the neutral model of evolution (*p* < 0.10) (Table 2). In addition to Tajima *D* values, the Fu’s *F*s statistic based on the distribution of haplotypes displayed minus values, indicating an excess of rare haplotypes over what would be expected under neutrality; especially *coxI* exhibited significant negative values of both Tajima *D* and Fu’s *F*s statistic (*p* < 0.10) (Table 2). Each base non-synonymous substitution was analyzed in regards to the compensation of physicochemical properties of amino acid replacement. The *cytb* gene was found to have two positions of hydrophobic amino acid replacement from I15V and L66V. In the case of *coxI*, there were five amino acid replacements in the *L. sabrazesi* population, including R6I, Y32C, K56N, S99T and A113V, while *Plasmodium* spp. were found to have 23 amino acid substitutions, including R6K, Y32K, R50 K, N58T, N58K, N61K, K66I, L67H, I71M, S73F, L74F, F81L, C93W, P95S, K97E, P99A, K102R, I103L, Q111H, G116E, L117F, F119I, P123A, S123A, F126C and F126Y. The *coxIII* gene was found to have six amino acid replacements, including T4H, T4P, L5I, L32I, S55F, I69T and I79S. However, all amino acid replacements exhibiting the most conservative replacements occurred by non-synonymous substitution.

### Haplotype diversity

**Fig. 7:**
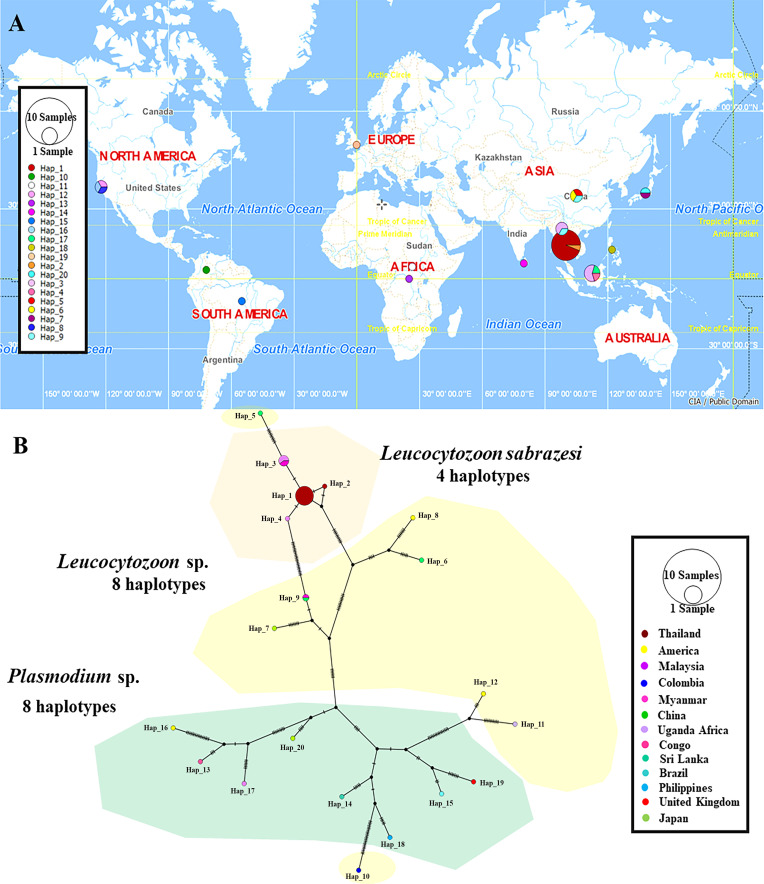
TCS network of haplotypes based on *Leucocytozoon* spp. and *Plasmodium* spp. *cytb* gene sequences (A) detected in Thailand and other countries. The number of bars on lines between a haplotype and another represent the number of nucleotide mutation (B).

**Fig. 8:**
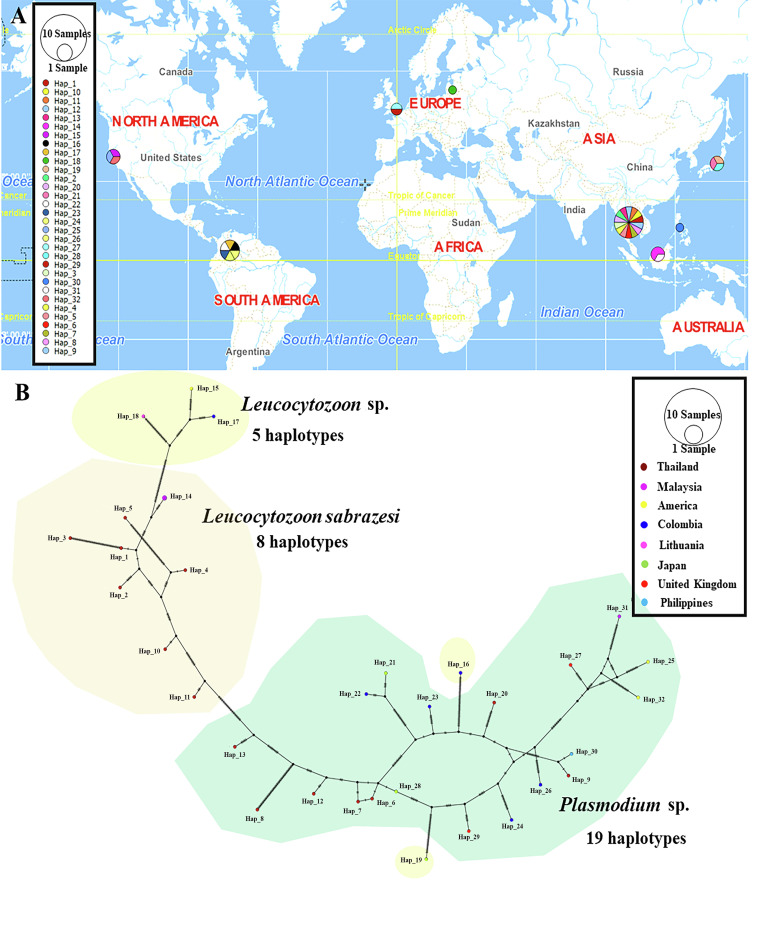
TCS network of haplotypes based on *Leucocytozoon* spp. and *Plasmodium* spp. *coxI* gene sequences (A) detected in Thailand and other countries. The number of bars on lines between a haplotype and another represent the number of nucleotide mutation (B).

**Fig. 9:**
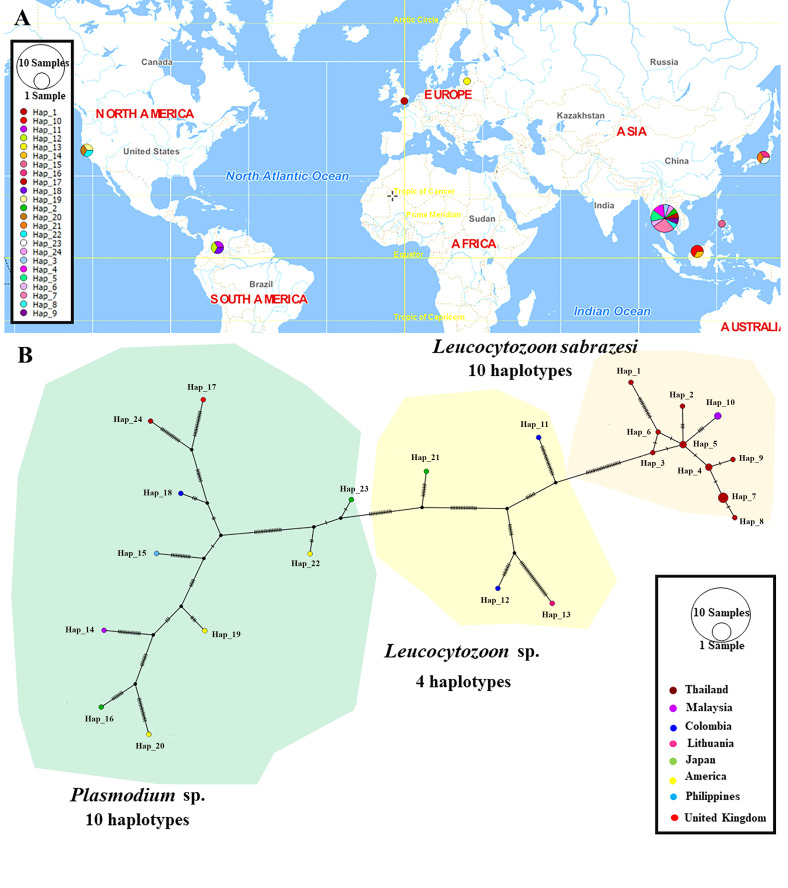
TCS network of haplotypes based on *Leucocytozoon* spp. and *Plasmodium* spp. *coxIII* gene sequences (A) detected in Thailand and other countries. The number of bars on lines between a haplotype and another represent the number of nucleotide mutation (B).

**Fig. 10:**
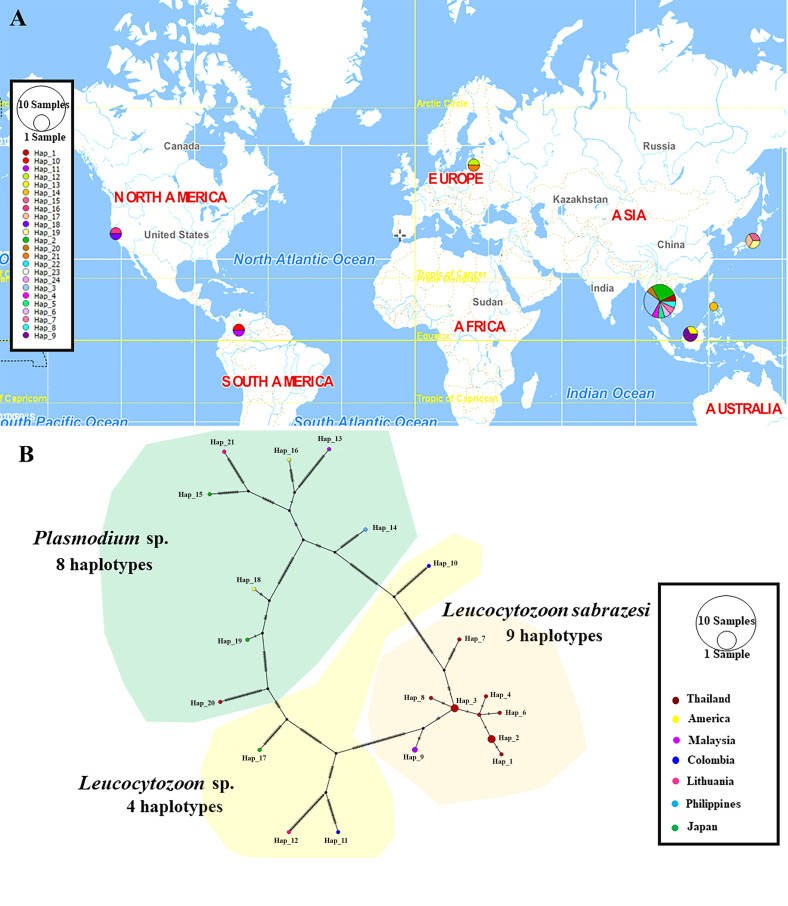
TCS network of haplotypes based on *Leucocytozoon* spp. and *Plasmodium* spp. concatenated gene sequences (A) detected in Thailand and other countries. The number of bars on lines between a haplotype and another represent the number of nucleotide mutation (B).

The TCS Network tool was used to construct the haplotype network of the *cytb, coxI* and *coxIII* gene sequences of *Leucocytozoon* spp. and *Plasmodium* spp. The haplotype of each gene was estimated together with geographic distribution, consistently displaying high variation from multiple sequence alignment. The *coxI* gene showed a greater number of nucleotide variations and higher diversity than *coxIII* and *cytb*. However, *L. sabrazesi* harbored 4, 8 and 10 haplotypes of *cytb, coxI* and *coxIII*, respectively. For *L. sabrazesi cytb* gene Thailand strain, our findings showed that most sequences are found in haplotype #1 and some sequences are found in haplotypes #3 and #4 obtained from Myanmar and Malaysia (Fig. 7, Tables 2 and 3). In the case of *coxI*, *L. sabrazesi* Thailand strain contained seven haplotypes, including haplotypes #1 to #5 and #10 to #11 formed the nearest clade with haplotype #14 of *L. sabrazesi* Malaysia strain. In addition to the *coxI* gene of *L. sabrazesi*, haplotype #9 of *P. gallinaceum* from Thailand formed the nearest branch to haplotype #30 of *P. gallinaceum* from the Philippines. Five haplotypes of *P. juxtanucleare* from Thailand, including haplotypes #6 to #8, #12 and #13 also formed the nearest branch to haplotype #28 of *P. juxtanucleare* from Japan (Fig. 8, Tables 2 and 3). Additionally, nine haplotypes of *L. sabrazesi coxIII* gene Thailand strain exhibited the nearest branch to haplotype #10 of *L. sabrazesi* Malaysia strain (Fig. 9, Tables 2 and 3). The concatenated gene comprising eight haplotypes in *L. sabrazesi* Thailand strain also grouped together with haplotype #9 in *L. sabrazesi* Malaysia strain (Fig. 10, Tables 2 and 3).

## Discussion

Leucocytozoonosis caused by the hemoprotozoan *L. sabrazesi* is an important insect-borne disease of chickens and causes high economic losses to chicken industries worldwide, including in Thailand. In general, genetic diversity is a survival strategy which is employed by parasites to evade the immune responses of avian hosts (chickens, ducks and birds) [5, 37]. There have been studies of genetic diversity of *Leucocytozoon* sp. based on the mitochondrial gene sequences in several countries, and almost all of these studies focused on the *cytb* gene [8, 18, 29, 44]. However, there has been no information available regarding the genetic diversity and phylogeny of *L. sabrazesi* mitochondrial genes in Thailand until now. In the present study, we used the *cytb, coxI, coxIII* and concatenated genes in the chicken population sampled in Thailand to ascertain the genetic diversity of *L. sabrazesi* and their co-infections in these regions.

The molecular detection and DNA sequencing displayed the highest similarity of both *cytb* and *coxIII* genes of *L. sabrazesi.* Interestingly, this is the first report of co-infection between *L. sabrazesi* and *P. gallinaceum* and that of *L. sabrazesi* and *P. juxtanucleare* in the leucocytes of chickens in Thailand*.* Notably, the *coxI* gene has the ability to cross-react and could be used to detect infection of *L. sabrazesi* and *Plasmodium* spp. Our findings are consistent with the report obtained by Pacheco et al. [32]. A phylogenetic analysis was carried out to display the relationship between individual and multi-locus genes of mitochondria determining the detection of *L. sabrazesi*. Moreover, the *coxI* gene has been employed to detect te infection of *P. gallinaceum* and *P. juxtanucleare* in chickens from Bongti and Tha Sao districts in Kanchanaburi province located near the Chacheongsao province of Thailand which are reported about *P. gallinaceum* [34] and near at the border of Myanmar which are reported regarding *P. juxtanucleare* in chickens [44]. Regarding three mitochondrial nucleotide sequences, our results indicated the highest sequence similarity to *L. sabrazesi* and some co-infected with *P. gallinaceum* and *P. juxtanucleare.*

Genetic variation of three mitochondrial genes commonly occurred in *Plasmodium* spp., while *coxI* showed high genetic variation in *Leucocytozoon* spp. However, these genes were found to have higher transition than transversion rates, and caused mutational bias to high A-T content and were proned to express the evolutionary saturation for divergence of parasites, which are consistent with the analysis of hemosporidian mitochondrial genomes [33]. Moreover, the lack of mitochondrial sequences from *Leucocytozoon* spp. and *Plasmodium* spp. directly affected the evolutionary analysis. These genes displayed *K*_a_/*K*_s_ ratios less than one and minus values, indicating purifying selection [30]. Tajima’s *D* results indicated minus values, but only *coxI* indicated selective sweep, which was consistent with the negative value of Fu’s *F*s statistic which determined the population expansion under statistical significance [15]. In addition, the *cytb* and *coxIII* genes indicated minus values of *K*_a_/*K*_s_ ratios that determined purifying selection, but both Tajima’s *D* and Fu’s *F*s were negative and not significant, indicating neutrality or perhaps these values can result in indirect selection from balancing selection on a nearby locus (linked genes) [38]. All evolutionary analyses reflected that hemosporidian organisms passed through the important obstacle of evolution like genetic drift before performing population expansion later [7, 14]. In addition, some variations affected haplotype distribution, which occurred from the polyphyletic relationship of genus *Leucocytozoon* spp. and likely displayed as an ancestor of avian parasites [27]. In addition, only partial nucleotide sequences exhibited the number of synonymous greater than non-synonymous substitution, and amino acid replacement caused by non-synonymous substitution did not show lethal effects to *L. sabrazesi* and mitochondrial genome variation caused by the host switching during their life cycle [33]. However, the number of non-synonymous substitutions affecting amino acid replacements exhibited a higher number of conservative than radical amino acid replacements, reflecting the purifying selection of mitochondrial genes [11]. In addition to nucleotide substitution, the non-synonymous substitutions which caused the amino acid substitutions were estimated concerning the compensation of amino acids by physicochemical properties through PROVEAN program. We found that all amino acid substitutions did not affect their function based on comparisons from the NCBI database. Moreover, the multiple amino acid sequence alignment also consistently displayed compensation of physicochemical properties of amino acid replacement through BLOSUM 62 score and exhibited a higher number of conservative than radical amino acid replacements with the dark blue color (Supplementary Figs. 1B, 2B, 3B).

In this study, the entropy and multiple sequence analysis showed the genetic variation in *Plasmodium* spp. to be greater than in *Leucocytozoon* spp. This indicated greater diversification of malaria parasites and the paraphyletic relationship among avian hemosporidians [31]. However, *Leucocytozoon* spp. displayed a higher number of haplotypes than *Plasmodium* spp., these values were affected in populations of *Leucocytozoon* sp. but not *L. sabrazesi* [37]*.* Similarly, haplotype diversity indicated the close genetic relationship among *L. sabrazesi.* detected in Thailand, Malaysia and Myanmar [44].

## Conclusions

This study is the first report on the genetic diversity of *L. sabrazesi* based on the mitochondrial genes including *cytb, coxI, coxIII* and concatenated sequences in Thailand. The co-infection between *L. sabrazesi* either *P. gallinaceum* or *P. juxtanucleare* in chickens in Thailand was investigated. The advantage of cross-PCR amplification of the *coxI* gene is that it can discriminate co-infection, which is not verified by microscopic examination. Even though the phylogenetic relationship and evolutionary distribution showed high genetic variation and haplotype diversity in the *coxI, coxIII* and *cytb* genes, they still indicated purifying selection, which occurred together with population expansion after genetic drift events in switching-host hemosporidian populations. These findings could help to improve the understanding of molecular phylogenetics and diversity among these mitochondrial sequences of *L. sabrazesi* Thailand strain. Our findings could therefore be beneficial for the development of immunodiagnostic tools and vaccine strategies for chicken leucocytozoonosis.

## Supplementary materials

The Supplementary materials of this article are available at https://www.parasite-journal.org/10.1051/parasite/2022022/olm

*Table S1:* Similarity of the *cytb* gene sequences of *Leucocytozoon* spp. and *Plasmodium* spp. as detected in chicken samples in Thailand compared with other sequences taken from GenBank. *Table S2:* Similarity of the *cox I* gene sequences of *Leucocytozoon* spp. and *Plasmodium* spp. as detected in chicken samples in Thailand compared with other sequences obtained from GenBank. *Table S3:* Similarity of the *cox III* gene sequences of *Leucocytozoon* spp. and *Plasmodium* spp. as detected in chicken samples in Thailand compared with other sequences taken from GenBank. *Table S4:* Similarity of the *cytb* amino acid sequences of *Leucocytozoon* spp. and *Plasmodium* spp. as detected in chicken samples in Thailand compared with other sequences taken from GenBank. *Table S5:* Similarity of the *cox I* amino acid sequences of *Leucocytozoon* spp. and *Plasmodium* spp. as detected in chicken samples in Thailand compared with other sequences taken from GenBank. *Table S6:* Similarity of the *cox III* amino acid sequences of *Leucocytozoon* spp. and *Plasmodium* spp. as detected in chicken samples in Thailand compared with other sequences taken from GenBank.
